# A Comparison of the Nutritional and Biochemical Quality of Date Palm Fruits Obtained Using Different Planting Techniques

**DOI:** 10.3390/molecules26082245

**Published:** 2021-04-13

**Authors:** Jeerawan Hinkaew, Amornrat Aursalung, Yuraporn Sahasakul, Nattapol Tangsuphoom, Uthaiwan Suttisansanee

**Affiliations:** 1 Institute of Nutrition, Mahidol University, Salaya, Phuttamonthon, Nakhon Pathom 73170, Thailand; ccindy.jee@hotmail.com (J.H.); amornrat.aur@mahidol.ac.th (A.A.); yuraporn.sah@mahidol.ac.th (Y.S.); nattapol.tng@mahidol.ac.th (N.T.); 2 Food and Nutrition Academic and Research Cluster, Institute of Nutrition, Mahidol University, Salaya, Phuttamonthon, Nakhon Pathom 73170, Thailand

**Keywords:** *Phoenix dactylifera* L., cell culture origin, seed origin, nutritional compositions, phenolics, carotenoids, in vitro health properties

## Abstract

Date palm fruit (*Phoenix dactylifera* L.) is commonly consumed around the world and has recently become an economical crop in Eastern Thailand, especially the Barhi cultivar that can be consumed as fresh fruit. To maintain genetic qualities, date palm is populated through cell culture. This leads to high production costs, while access to this technique is limited. Increasing date palm population by simple seed planting is currently of interest as an alternative for local farmers. Nevertheless, information on nutritive values, bioactive compounds, and health-promoting bioactivities of seed originating from date palm fruit is unavailable. Effects of different planting origins (cell culture origin (CO) and seed origin (SO)) of date palm fruits at the Khalal stage of Barhi cultivar were investigated for nutritive values, bioactive compounds, and in vitro health-promoting properties via key enzyme inhibitions against obesity (lipase), diabetes (α-amylase, α-glucosidase, and dipeptidyl peptidase-IV), Alzheimer’s disease (cholinesterases and β-secretase), and hypertension (angiotensin-converting enzyme). Waste seeds as a by-product from date palm production were also examined regarding these properties to increase seed marketing opportunities for future food applications and other health-related products. CO and SO exhibited insignificant differences in energy, fat, and carbohydrate contents. SO had higher protein, dietary fiber, vitamin A, vitamin E, and calcium contents than CO, while CO contained higher contents of fructose, glucose and maltose. Higher phenolic contents in SO led to greater enzyme inhibitory activities than CO. Interestingly, seeds of date palm fruits mostly contained higher nutritive values than the flesh. No carotenoids were detected in seeds but higher phenolic contents resulted in greater enzyme inhibitory activities than recorded for fruit flesh. Results suggest that appropriate planting of date palm can support the development of novel date palm fruit products, leading to expansion of economic opportunities and investment in date palm fruit agriculture.

## 1. Introduction

Date palm (*Phoenix dactylifera* L.) is grown as a commercial agricultural plant in the Middle East and North Africa with more than 1500 varieties [1,2]. Date palm fruits are commonly consumed around the world, especially in Arabian regions such as Saudi Arabia, Iran, Egypt, and Algeria [3]. Date palm fruit development can be divided into five stages as Hanabauk, Kimri, Khalal, Rutab, and Tamr [4], with observation of various physical properties including size, shape, color, texture, and flavor [5]. Date palm fruits at the last three stages (Khalal, Rutab, and Tamr) are commonly consumed due to their soft texture and sweet flavor. Dry date palm fruits at the Tamr stage are popular and available in markets during all seasons. However, fresh date palm fruits at the Khalal and Rutab stages of particular cultivars are currently of interest, since they can be consumed in fresh form. Fruits of Khalas, Medjool, and Deglet Nour cultivars are generally consumed in dry form, while Barhi cultivar fruits are consumed fresh. Interestingly, nutrients, volatile substances, bioactive compounds, and functional properties of dry and fresh date palm fruits vary according to both internal and external factors such as postharvest management, cultivars, stages of maturity, and growing environment [6,7,8,9,10].

Date palm fruit at the Khalal stage of Barhi cultivar is a significant source of nutrients and bioactive compounds with advantageous health benefits [2,10]. This cultivar has become an economical crop in Eastern Thailand. Date palm is normally multiplied using the cell culture technique to sustainably maintain the genetics of the exact cultivar and control fruit quality (physical appearance (i.e., size and color), taste, and flavor). However, this technique requires botanical specialists, leading to high costs with limited accessibility for local farmers. Therefore, a simple seed planting technique to reduce cost with easy access is a preferred alternative for local production. This technique might induce genetic variation because of natural selection but offers an excellent chance for date palm to self-adapt to the environment (i.e., insect and drought tolerance). Genetic variation might also cause alteration in nutrients and bioactive compounds. Some changes can be detrimental, whereas others may improve traits of the original cultivar. However, information comparing cell culture and seed originating from date palm fruits regarding their nutrients, bioactive compounds, and in vitro health properties is currently unavailable.

The flesh of date palm fruit is the only edible part, while seeds as the major waste product of date palm production, are generally used as ingredients to increase nutrients in animal feed due to their high fiber, potassium, and magnesium contents [11]. A previous study stated that date palm seeds could be used as non-caffeinated coffee [12]. Interestingly, previous researches indicated that date palm seeds exhibited high values of total phenolic contents (TPCs) [13] and antioxidant activity determined by oxygen radical antioxidant capacity (ORAC) assay [14]. Date palm seeds were also reported to provide higher TPCs and antioxidant activity than the flesh [15]. Therefore, date palm seeds have attracted interest for development as a by-product containing valuable bioactive compounds and as a potential food application. However, data concerning nutritive values, bioactive compounds, and in vitro health properties of date palm fruit and seed grown in Thailand are limited.

Therefore, here, cell culture and seed originating from date palm fruits at the Khalal stage of Barhi cultivar were investigated regarding their nutritional values, bioactive compounds (phenolic acids, flavonoids, and carotenoids) and in vitro health properties by targeting inhibitions of the key enzymes that control some non-communicable diseases including obesity (lipase), diabetes (α-amylase, α-glucosidase, and dipeptidyl peptidase-IV), Alzheimer’s disease (acetylcholinesterase, butyrylcholinesterase, and β-secretase), and hypertension (angiotensin-converting enzyme). Different date palm fruit parts including flesh and seeds at the Khalal stage of Barhi cultivar were also investigated. Results can be used to promote appropriate agriculture to increase the consumption of date palm as a healthy fruit and support the development of novel date palm fruit products, leading to the expansion of economic opportunities and investment in date palm fruit agriculture

## 2. Results

### 2.1. Sample Characterization

Physical appearances of cell culture (CO) and seed (SO) originating from date palm fruits at the Khalal stage of Barhi cultivars are shown in Supplementary Materials (Tables S1 and S2). CO varieties contain 14–15 fruits in each bunch, while SO varieties contain 21–29 fruits. A whole date palm fruit was separated into the flesh (edible part composed of epicarp (outer skin) and mesocarp), and seed. Flesh of the CO fruits was significantly longer (approximately 3.73 cm) than flesh of SO fruits (approx. 3.13 cm), with insignificant differences in width (2.00–2.11 cm) and thickness (0.54–0.67 cm). The CO seeds were also significantly longer (approx. 2.55 cm) than SO seeds (approx. 2.14 cm), while the latter were significantly wider (approx. 0.88 cm) than the former (approx. 0.82 cm). Both seed types showed insignificant differences in thickness (approx. 0.61–0.64 cm).

Color measurement of fresh and dry forms of flesh and seed of CO and SO fruits was expressed in CIELAB units, in which L* represents black (0) to white (100) colors, a* represents green (−) to red (+) colors, and b* represents blue (−) to yellow (+) colors as shown in Supplementary Table S3. Color analysis suggested that the epicarp of fresh SO exhibited significantly higher b* value (yellower) than that of CO, while insignificant differences in L* and a* values were observed. The freeze-drying process increased the L* value but decreased a* and b* values in all samples, suggesting that fresh samples had a darker yellow color than dried samples. Fresh SO seed exhibited significantly higher a* and b* values than fresh CO seed, while insignificant differences in L* values were observed. These results suggested that fresh SO seed was a more reddish yellow color than CO seed. After the freeze-drying process, all dry samples exhibited increased b* value and decreased a* value but insignificantly different L* values, suggesting that dry samples were a lighter yellow color than their corresponding fresh counterparts. The seed showed significantly higher a* and b* values but lower L* values than the flesh, suggesting that the seed exhibited a darker reddish yellow color than the flesh.

Moisture contents were measured in both fresh and dry samples as shown in Supplementary Table S3. Results suggested that fresh flesh of SO fruits exhibited significantly higher moisture content (75.68%) than CO fruits (68.98%). Moisture content of fresh fruit flesh was 1.6–2.1 times higher than fresh seeds, while moisture contents of CO and SO seeds were insignificantly different (34.42–36.23%). After freeze drying, the moisture contents were reduced to less than 10% (2.93–3.57%), which prevented microbial growth.

### 2.2. Nutritive Values

Nutritive values of different originated date palm fruits per 100 g dry weight (DW) are shown in Table 1. Insignificant differences in energy and fat contents were observed between CO and SO; however, flesh of SO possessed significantly higher protein content than flesh of CO varieties. Protein and fat were detected in low amounts, accounting for only 3.67–4.82% of total energy. Therefore, the main component providing energy was carbohydrate, ranging between 91.64 and 93.47 g. Dietary fiber, composed of soluble dietary fiber (SDF) and insoluble dietary fiber (IDF), in flesh of SO was significantly higher than that of CO, while IDF content was approximately five times higher than SDF. On the other hand, flesh of CO exhibited significantly higher total sugar content than flesh of SO. Four types of sugar were detected in flesh of CO including fructose, glucose, maltose and sucrose, while only the first two were detected in flesh of SO. Considering fat-soluble vitamins, vitamin A and vitamin E contents in flesh of SO were significantly higher than those in CO. Vitamin D was lower than the detectable limit (0.05 µg/100 g fresh weight (FW)), while vitamin C was undetected in this study. Furthermore, significantly higher contents of minerals including Ca, P, Na, Mg, and Zn were recorded in SO than in flesh of CO, while flesh of CO exhibited significantly higher Fe contents than SO. However, K contents were insignificantly different between flesh of CO and SO.

When comparing nutritive values (per 100 g DW, Table 1) between different originated date palm seeds, CO seeds exhibited significantly higher energy and fat contents than SO seeds, whereas protein content in SO seeds was significantly higher than in CO seeds. Protein and fat were detected in low amounts (only 21.70–23.27% of total energy). The main component providing energy was carbohydrate, with insignificantly different contents observed between CO and SO seeds. Total dietary fibers, along with both soluble and insoluble forms, were insignificantly different. IDF content was approximately 80 times higher than SDF, while insignificant differences in fructose and glucose contents were detected between CO and SO seeds. For fat-soluble vitamins, CO and SO seeds exhibited insignificantly different vitamin A and vitamin E contents. Similar to their flesh contents, vitamin D was lower than the detectable limit (0.05 µg/100 g FW) and vitamin C was undetected in both CO and SO seeds. Furthermore, insignificant differences in minerals including P, Na, K, Fe, and Zn were observed between CO and SO seeds. However, Ca and Mg contents in SO seeds were significantly higher than in CO seeds.

Comparison of nutritive values between flesh and seeds of CO suggested that flesh provided significantly higher carbohydrate, SDF, ash, sugar, vitamin A and vitamin E contents, along with minerals including Ca, Na, and K than seeds. However, seeds of CO provided significantly higher energy, protein, fat, total dietary fiber and IDF contents, along with minerals including P, Mg, Fe, and Zn than the corresponding flesh. Similar results were observed for flesh and seeds of SO; however, insignificant differences in P and Zn contents were detected.

### 2.3. Total Phenolic Contents, Total Flavonoid Contents, Phenolic and Carotenoid Profiles

Results suggested that both flesh and seeds of SO exhibited higher total phenolic contents (TPCs) than CO (Table 2). Similar results were observed for total flavonoid contents (TFCs), in which both flesh and seeds of SO exhibited higher TFCs than CO. When considering the same cultivated origin, TPCs and TFCs of seeds were significantly higher than those of flesh in both CO and SO varieties.

Phenolic profiles (Table 2) indicated that flesh of both CO and SO contained phenolic acids including ferulic acid, vanillic acid, syringic acid, sinapic acid, *p*-coumaric acid, and caffeic acid. Comparing different cultivar origins, flesh of CO contained significantly higher contents of syringic acid and *p*-coumaric acid than flesh of SO, while the latter exhibited significantly higher contents of vanillic acid, caffeic acid, ferulic acid, and sinapic acid than the former. Results also indicated that flesh of CO and SO contained five flavonoids including isorhamnetin, hesperidin, kaempferol, luteolin, and apigenin. Comparing different cultivar origins, flesh of CO exhibited significantly higher hesperidin, kaempferol, and apigenin contents than flesh of SO. However, the latter provided significantly higher luteolin and isorhamnetin contents than the former. For carotenoids, flesh of SO contained higher lutein, β-cryptoxanthin, α-carotene, and β-carotene contents than flesh of CO.

Phenolic acid contents in seeds were similar to those found in flesh, including vanillic acid, caffeic acid, syringic acid, *p*-coumaric acid, ferulic acid, and sinapic acid, while gallic acid was only found in seeds. When comparing different cultivar origins, seeds of CO exhibited significantly higher vanillic acid, syringic acid, ferulic acid, and sinapic acid contents than seeds of SO. However, insignificant differences in gallic acid, caffeic acid, and *p*-coumaric acid contents were observed in seeds of CO and SO. Besides phenolic acids, seeds of CO and SO also contained flavonoids including luteolin, quercetin, kaempferol, apigenin, and isorhamnetin. Seeds of SO contained higher contents of luteolin, quercetin, kaempferol, and isorhamnetin than seeds of CO, while insignificant differences in apigenin contents were observed, and no carotenoids were detected in seeds of both CO and SO.

When comparing flesh and seeds of CO, results suggested that flesh contained significantly higher *p*-coumaric acid, ferulic acid, sinapic acid, kaempferol, apigenin, and isorhamnetin contents than seeds, while vanillic acid, caffeic acid, and luteolin contents were significantly higher in seeds than in flesh. Nevertheless, insignificant differences in syringic acid and hesperidin contents were observed in flesh and seeds. Similar results were observed between flesh and seeds of SO, with the exception of luteolin, where significantly higher content was detected in flesh than in seeds. Gallic acid and quercetin of both CO and SO were only detected in seeds, while hesperidin and carotenoids were only found in flesh.

### 2.4. Enzyme Inhibitory Activities

One of the key enzymes involved in the control of obesity is lipase, a lipid degrading enzyme. The inhibitory activities of lipase in CO and SO ranged 4.19–77.14% using an extract concentration of 2.5 mg/mL (Table 3). Comparing different cultivar origins, flesh of CO exhibited significantly higher lipase inhibitory activity than flesh of SO. However, opposite results were observed in seeds. Seeds of SO exhibited significantly higher lipase inhibitory activities than seeds of CO. Interestingly, seeds exhibited significantly higher lipase inhibitory activities than flesh in both CO and SO.

The key enzymes involved in diabetes are α-amylase, α-glucosidase, and dipeptidyl peptidase IV (DPP-IV). The first two are carbohydrate degrading enzymes, while the last is involved with the release of glucagon (inhibition) and insulin secretion (stimulation); thus, inhibition of these enzymes lowers blood glucose. Results suggested that only seeds exhibited α-amylase inhibitory activities in the range of 63.51–70.14% using extract concentration of 25 mg/mL, while no inhibition was observed in flesh using the same extract concentration (Table 3). Comparing different cultivated origins, seeds of SO exhibited significantly higher α-amylase inhibitory activities than seeds of CO. For α-glucosidase inhibitions, flesh of CO and SO exhibited inhibitory activities in the range of 44.64–50.52% using extraction concentration of 2.5 mg/mL, while their seeds exhibited inhibitory activities in the range of 52.33–60.38% using extraction concentration of 0.075 mg/mL (Table 3). In both flesh and seeds, CO exhibited significantly higher α-glucosidase inhibitory activities than SO. Even though statistical analysis cannot be applied to compare between flesh and seeds of the same cultivated origin (different extract concentrations), low extract concentration of seeds (0.075 mg/mL) used in the enzyme assay yielded higher inhibitory activities than flesh, with higher extract concentration (2.5 mg/mL). This result suggested that seeds were more effective inhibitors against α-glucosidase than flesh. Nevertheless, low inhibitory activity against DPP-IV was only observed in flesh of SO at 4.39% inhibition using extract concentration of 12.5 mg/mL (Table 3); thus, DPP-IV inhibition might not be an effective target to control diabetes using date palm fruit.

Acetylcholinesterase (AChE), butyrylcholinesterase (BChE), and β-secretase (BACE-1) are the key enzymes involved in controlling Alzheimer’s disease (AD) pathology. The first two are neurotransmitter degrading enzymes, while the last involves β-amyloid formation. AChE inhibitory activity in flesh was only detected in SO at 23.49% inhibition using extract concentration of 20 mg/mL, while none was detected in CO (Table 3). However, seeds of both CO and SO inhibited AChE in the range 49.49–67.97% using extract concentration of 2 mg/mL. Comparing different cultivated origins, seeds of CO exhibited significantly higher AChE inhibitory activity than seeds of SO. Similar to AChE inhibition, BChE inhibitory activity was only detected in flesh of SO (31.20% inhibition using extraction concentration of 20 mg/mL), while none was detected in flesh of CO (Table 3). However, seeds exhibited BChE inhibitory activities ranging from 27.19 to 46.80% using extract concentration of 0.2 mg/mL. Seeds of CO exhibited significantly higher BChE inhibitory activity than seeds of SO. Even though statistical analysis cannot be applied to compare flesh and seeds of the same cultivated origin (different extract concentrations), low extract concentration of seeds (2 mg/mL in AChE assay and 0.2 mg/mL in BChE assay) used in the enzyme assay yielded higher inhibitory activities than flesh at higher extract concentration 20 mg/mL. These results suggested that seeds were more effective inhibitors than flesh in both AChE and BChE reactions. Inhibitory activities against BACE-1 of date palm flesh ranged 70.73–75.05% using extract concentration of 20 mg/mL (Table 3). Comparing different cultivated origins, flesh of CO exhibited significantly higher BACE-1 inhibitory activity than flesh of SO. Unlike flesh, seeds exhibited lower BACE-1 inhibitory activities, ranging 24.50–39.23% using the same extract concentration as flesh (20 mg/mL). Seeds of CO exhibited significantly higher BACE-1 inhibitory activity than seeds of SO, while in both CO and SO, flesh exhibited higher inhibitory activity than seeds.

One of the key enzymes involved in hypertension is angiotensin-converting enzyme (ACE). This enzyme degrades angiotensin I into angiotensin II, resulting in increased blood pressure. Inhibitory activities against ACE of date palm flesh ranged 20.68–85.07% using extract concentration of 0.55 mg/mL (Table 3). Flesh of SO exhibited significantly higher ACE inhibitory activity than flesh of CO. Likewise, seeds exhibited ACE inhibitory activities ranging 58.69–61.36% at the same extract concentration as flesh. However, insignificant differences in ACE inhibitory activities were observed between CO and SO flesh. Comparing different fruit parts, seeds of CO exhibited significantly higher ACE inhibitory activities than flesh, with an opposite result observed for SO, where flesh exhibited significantly higher ACE inhibitory activities than seeds

## 3. Discussion

This research found that compared to CO; (i) SO exhibited higher nutritive values including protein, fiber, vitamins (A and E), and minerals (Ca, P, Na, Mg, and Zn) with lower sugar and total carbohydrate contents, (ii) SO exhibited higher bioactive compounds, especially carotenoids, (iii) SO exhibited greater cholinesterase and angiotensin-converting enzyme inhibitory activities, while differences in other enzyme inhibitory activities between CO and SO were within ± 5% inhibition, and (iv) date palm seeds exhibited higher nutritive values including protein, fat, fiber, phenolic contents and most health-promoting bioactivities but showed lower sugar contents than flesh.

### 3.1. Nutritive Values of Date Palm Flesh

Our results indicated that date palm flesh contained high energy and nutritional values (reducing sugar, dietary fiber, vitamins, and minerals), with SO exhibiting higher protein, fiber, vitamins (A and E), and most mineral contents than CO, while the latter contained higher sugar and carbohydrate contents. Compared to results in the literature, nutritive values of CO and SO flesh, i.e., protein and ash levels were higher, while fat, carbohydrate, and energy levels were slightly lower than those of date palm flesh in the United Arab Emirates (UAE) [6]. Only nutritive values for date palm flesh in the Tamr stage in Barhi cultivar were available, and results reported lower total dietary fiber than our CO and SO [16]. Interestingly, date palm flesh of offshoot origin at the Khalal stage of Khlass cultivar exhibited higher crude fiber than its cell culture origin [17]. These results were in good agreement with our report. Previously, insoluble dietary fiber (IDF) was suggested as a major component of date palm total fiber flesh in Deglet-Nour and Allig cultivars [18]. A similar trend was found in our study, where IDF was observed as a major dietary fiber in date palm flesh for both CO and SO. The recommended daily intake of fiber is 25 g/day; thus, our date palm fruits with approximately 5 g dietary fiber/100 g fresh weight (FW) can be considered as a good source of dietary fiber. IDF can be fermented by gut microbiota to produce short chain fatty acids which, in turn, promote gut-health, leading to bulk satiety and increased stool weight, while regular consumption of soluble dietary fiber (SDF) helps to reduce blood cholesterol and blood pressure [19,20,21]. Major components of sugar in CO and SO are glucose and fructose, with glucose to fructose ratios of 1.0–1.1. These results were in good agreement with a previous report, indicating that date palm flesh at the Khalal stage in Barhi cultivar contained approximately 1:1 glucose to fructose ratio [6]. Vitamin E was previously reported to be the major content in date palm flesh in the Sukkari cultivar, followed by vitamin Bs as minor components and non-detected vitamin A [22]. However, vitamin B-complex and C are also major vitamins in dried date fruits [1]. By contrast, our results suggested vitamin A as a major vitamin, followed by vitamin E. For element contents, our results were in good agreement with previous reports [6,23,24] that indicated K as the major element in date palm flesh. Amounts of K, P, and Ca in our research were higher, while Na content was much lower than previous results in the literature reported for the same stage and cultivar [6]. Variations in nutritional compositions of date palm flesh result from differences in varieties, maturation stages, geographic locations, cultivation conditions, agroclimatic and environmental conditions, and both pre and postharvest treatments [24].

### 3.2. Bioactive Compounds of Date Palm Flesh

Previous reports also indicated that date palm fruits in the Khalal stage of Akhrot, Hillawi-I, Qantar, Makran, Chohara, Kokna, Danda, and Shamran-I cultivars exhibited higher TPCs (3.49–5.71 mg GAE/g FW) than at the Rutab and Tamr stages of the same cultivar [25]. Similar results were observed in Zerin, Jaman, Pela Dora, Rachna, Seib, Zardo, Shado, Peli Sunder, Wahan Wali, and Champa Kali cultivars, in which date palm fruits in the Khalal stage exhibited higher TPCs (3.48–5.70 mg GAE/g dry weight (DW)) than for Rutab and Tamr stages of the same cultivar [26]. Compared to our TPC values (2.59–3.94 mg GAE/g DW), both CO and SO fruits exhibited TPCs similar to those previously reported. Differences in TPC values occurred from disparities in genetic variation, growth environment (location and climate), and extraction methods. Considering types of phenolics, the flesh of both CO and SO contained phenolic acids including ferulic acid, vanillic acid, syringic acid, sinapic acid, *p*-coumaric acid, and caffeic acid. A previous study reported that date palm fruit at the Khalal stage of Barhi balade cultivar contained gallic acid, 4-hydroxybenzoic acid, vanillic acid and ferulic acid, while caffeic acid and syringic acid were not detected (even though the standards were present in high performance liquid chromatography (HPLC) analysis) [10]. Syringic acid was, however, detected in the Tamr stage of Barhi balade cultivar, while caffeic acid was detected in the Rutab stage of Barhi iraqi cultivar [10]. Interestingly, sinapic acid was only reported in our research. Nevertheless, other phenolic acids reported in previous literature but not in our report (even though the standards for HPLC analysis were present in our study), included gallic acid and 4-hydroxybenzoic acid. No flavonoids were previously reported in date palm fruit at the Khalal stage of Barhi cultivar; however, the Tamr stage of Nabot Saif, Rashodia, Ajwa Al Madinah, Khodry, Khlas Al Ahsa, Sokary, Saffawy, Khlas Al Kharj, Mabroom, Khlas Al Qassim, Nabtit Ali and Khals El Shiokh cultivars were reported to contain quercetin, luteolin, apigenin, isoquercetin, and rutin [27]. Our results indicated that the flesh of CO and SO contained five flavonoids including isorhamnetin, hesperidin, kaempferol, luteolin, and apigenin, while quercetin, rutin, and isoquercetin were not observed in our study, even though the first two were used as standards in our HPLC analysis. Carotenoids were the most abundant bioactive compounds found in date palm fruit. Carotenoids in date palm fruit at the Khalal stage of Deglet-Nour, Tantebouchte, and Hamray cultivars included lutein and β-carotene [28], while lutein, β-cryptoxanthin, α-carotene, and β-carotene were present in CO and SO. Missing carotenoids reported in the literature were due to limitation in the standards used in HPLC analysis. Moreover, variation in maturity stage and cultivars, as well as other external factors (i.e., place of origin, extraction condition, and identified method) led to differences in bioactive compounds detected in date palm flesh.

### 3.3. Medicinal Properties of Date Palm Flesh

Medicinal properties through inhibition of enzymatic reactions related to non-communicable diseases including obesity (lipase), diabetes (α-amylase, α-glucosidase, and dipeptidyl peptidase-IV), Alzheimer’s disease (acetylcholinesterase, butyrylcholinesterase, and β-secretase), and hypertension (angiotensin-converting enzyme) of SO and CO were also investigated.

Incidence of overweight and obesity can be controlled by lipase, a fat degrading enzyme. Our results indicated that CO and SO flesh inhibited lipase, but degree of inhibition was low. The most abundant phenolic detected in date palm flesh as ferulic acid was previously reported to be a weak inhibitor against lipase, with half-maximal inhibitory concentration (IC_50_) of 5.03 mM [29], possibly leading to low lipase inhibitory activities in CO and SO flesh. The SO flesh exhibited higher TPCs than CO flesh and its lipase inhibitory activities were also greater.

Likewise, the key enzymes related to control of diabetes including α-glucosidase, α-amylase, and dipeptidyl peptidase IV (DPP-IV) were investigated. The first two are carbohydrate degrading enzymes; thus, they can retard the absorption of glucose into the bloodstream, thereby controlling diabetes, while DPP-IV inhibition promotes the release of insulin, resulting in low blood glucose levels. Ferulic acid inhibits α-glucosidase with an IC_50_ value of 1.13 mM [30], while lower inhibition was observed in α-amylase reaction (IC_50_ value 9.5 mM) [31]. Most phenolics strongly inhibit α-glucosidase, while lower inhibitory activities against α-amylase have also been observed [32,33]. This inhibitory effectiveness resulted in high α-glucosidase inhibitory activities for CO and SO flesh, while their α-amylase inhibitory activities were not detected. Moreover, sugar date palm fruit (*Phoenix sylvestris* (L.) Roxb.) extract inhibited α-glucosidase with an IC_50_ value of 3.18 µg/mL [34], suggesting the potential use of date palm fruit against diabetes through α-glucosidase inhibition. Similar results were observed with low DPP-IV inhibitory activity detected in SO flesh, while none was found in CO flesh, suggesting that phenolics might not act as effective DPP-IV inhibitors.

In Alzheimer’s disease (AD), two cholinesterases (ChEs) including acetylcholinesterase (AChE) and butyrylcholinesterase (BChE) are neurotransmitter degrading enzymes, while β-secretase (BACE-1) is involved with β-amyloid formation. Phenolics can act as strong ChE inhibitors [35,36], and higher TPCs were detected in SO flesh than in CO flesh. AChE and BChE inhibitory activities were detected in SO flesh, while no inhibitory activities were found in CO flesh. Interestingly, high BACE-1 inhibitory activities were observed in both SO and CO flesh. As well as phenolics that act as strong BACE-1 inhibitors (or non-peptidic inhibitors), short chain peptides (or peptidic inhibitors) also effectively inhibit BACE-1 activities [37,38]. Thus, high BACE-1 inhibitory activities might result from the biological functions of both phenolics and peptides in CO and SO flesh.

Interestingly, inhibition of the key enzyme in controlling hypertension, angiotensin-converting enzyme (ACE), in SO flesh was four times higher than in CO flesh, suggesting that strong inhibitors resided in SO flesh. Higher TPCs detected in SO flesh led to its significantly higher inhibitory activities. Ferulic acid is a weak ACE inhibitor with IC_50_ value of 4.4 mM [39]. As well as phenolics, short chain peptides also act as ACE inhibitors [40]. Thus, the high inhibitory activities observed in SO flesh might result from the bioactivities of its peptides rather than phenolics.

### 3.4. Nutritive Values, Bioactive Compounds, and Medicinal Properties of Date Palm Seeds

Date palm seeds are waste products from date palm processing industries, and commonly used as animal feed to increase fiber and mineral contents. Recently, date palm seeds have been used in food applications including non-caffeinated drinks and mayonnaise from date seed oil [11,12]. Most nutritive values of our date palm seeds concurred with a previous study that reported nutritive values of date palm seeds in 18 cultivars (including Barhi variety at the Tamr stage) [12], although Na content was higher in our study. Another study of date palm seed at the Tamr stage in Barhi cultivar reported 2–3 times higher Mg content and 7–9 times lower sugar contents compared to our of CO and SO seeds [41]. Differences in nutritional compositions of date palm seeds result from various factors including but not limited to variety, stage of maturation, growing region, environment, time of harvest and postharvest treatment [24].

Date palm seeds at the Tamr stage of Khalas and Sukkari cultivars exhibited TPCs of 20.14–20.60 mg GAE/g DW [42]. Likewise, date palm seeds at the Tamr stage of Khalas, Barhi, Lulu, Shikat alkahlas, Sokkery, Bomaan, Sagay, Shishi, Maghool, Sultana, Fard, Maktoomi, Naptit saif, Jabri, Kodary, Dabbas, Raziz, and Shabebe cultivars exhibited TPCs in the range of 18.64–47.68 mg GAE/g DW, with Barhi cultivar exhibiting the lowest [43]. Our date palm seeds at the Khalal stage of Barhi cultivar with TPCs of 10.76–18.41 mg GAE/g DW gave good agreement with these previous reports. Phenolic profiles of date palm seeds at the Tamr stage of Akerbouche, Tazizaout, Deglet-Nour, Ougherouss, Tantbouchte, Tafiziouine, and Tazerzait cultivars were also investigated. Some contained phenolic acids including cinnamic acid, coumaric acid, sinapic acid, and their derivatives [44]. Date palm seeds at the Tamr stage of Medjool cultivar also contained gallic acid, *p*-coumaric acid, caffeic acid, and syringic acid [45], while *p*-coumaric acid, ferulic acid, sinapic acid, and cinnamic acid derivatives were detected in date palm seeds in Akerbouche, Deglet-Nour, Ougherouss, Tantbouchte, Tafiziouine and Tazerzait cultivars [46]. Date palm seeds in Mabseeli cultivar were also reported to contain gallic acid, protocatechuic acid, *p*-hydroxybenzoic acid, vanillic acid, caffeic acid, *p*-coumaric acid, *m*-coumaric acid, *o*-coumaric acid, and ferulic acid [47]. However, little information on flavonoids was previously reported for date palm seeds. Specific flavonoids including catechin, proanthocyanins, and naringenin were reported in date palm seeds at the Tamr stage of Akerbouche, Tazizaout, Deglet-Nour, Ougherouss, Tantbouchte, Tafiziouine, and Tazerzait cultivars [44] and quercetin in date palm seeds of an unreported cultivar [48]. Unspecified flavonoids as flavones, flavonols, flavanones, and their glycoside derivatives were also reported [44,46]. Our CO and SO seeds contained similar phenolic acids (gallic acid, vanillic acid, caffeic acid, syringic acid, *p*-coumaric acid, ferulic acid, and sinapic acid) as these previous reports, while five flavonoids (luteolin, quercetin, kaempferol, apigenin, and isorhamnetin) were identified. No carotenoids were detected in our CO and SO seeds but trace amounts of lutein (0.07–0.27 mg/100 g FW), β-carotene (1.18–2.68 mg/100 g FW), α-carotene (0.00–0.08 mg/100g FW), γ-carotene (0.03–0.49 mg/100g FW), cryptoxanthin (0.03–0.15 mg/100g FW), and lycopene (0.00–0.03 mg/100 g FW) were previously reported in oil of date palm seeds in the Tamr stage of Khalas, Barhi, Lulu, Shikat alkahlas, Sokkery, Bomaan, Sagay, Shishi, Maghool, Sultana, Fard, Maktoomi, Naptit saif, Jabri, Kodary, Dabbas, Raziz, and Shabebe cultivars [49].

Date palm seeds exhibited higher TPCs than flesh in both CO and SO, while all seed samples demonstrated higher inhibitory activities against lipase, α-glucosidase, α-amylase, AChE, and BChE. Methanolic extracted date palm seeds in Ruchdi, Deglet Nour, Kentichi, and Ftimi cultivars inhibited pancreatic lipase with IC_50_ values ranging from 1.21 to 96.45 µg/mL [43], while consumption of date palm seed powder in Deglet Nour cultivar significantly reduced cholesterol and low-density lipoprotein (LDL) in rats induced with a high cholesterol diet [50]. For anti-diabetic properties, aqueous extracted date palm seeds in Fardh, Naghal, Khalas, Khinaizi, and Khasab cultivars inhibited α-glucosidase activities in the range 34.46–51.71% using extract concentration of 5 mg/mL [51]. The same seed extracts also inhibited α-amylase in the range 13.71–51.45% using the same extraction concentration as α-glucosidase inhibitory assay [51]. Date palm seed extracts also expressed in vivo anti-diabetic properties on streptozotocin- and alloxan-induced diabetic rats [47,52]. Phenolics in date palm seeds were suggested to be responsible for these activities [47,52]. Nevertheless, information on anti-AD properties via inhibition of AChE and BChE activities in date palm seeds has not yet been reported, even though our results indicated that their inhibitions were significantly higher than those of date palm flesh. These inhibitory activities resulted from the biological functions of phenolics detected in higher amounts in date palm seeds than in flesh. However, BACE-1 and ACE inhibitory activities of seeds were lower than in flesh under the same extract concentration (with the exception of CO seeds that exhibited higher ACE inhibitory activity than its flesh). Date palm flesh might contain particular phenolics or short chain peptides that are specific to these enzymes, leading to stronger enzyme-inhibitor binding and, thus, greater inhibitory activities. A previous study also stated that protein hydrolysates from date palm seeds exhibited ACE inhibitory activity with IC_50_ value of 0.5–1 mg/mL [53], suggesting potential anti-hypertensive properties through ACE inhibition of date palm seeds.

## 4. Materials and Methods

### 4.1. Sample Collection, Preparation, and Extraction

Date palm fruits (*Phoenix dactylifera* L.) at Khalal stage of Barhi cultivar including cell culture and seed origins (CO and SO, respectively) were supplied by T.A.P. Chon Buri Co., Phanat Nikhom district, Chon Buri Province, Thailand. SO was obtained from date palms growing from CO seed. Both samples were collected during July to August, 2019 from Bo Phloi District, Kanchanaburi Province, Thailand (14°17’58.8”N and 99°28’40.8”E). The voucher specimens, including BK No. 071406 (CO) and BK No. 071407 (SO), were deposited at the Bangkok Herbarium (BK), Bangkok, Thailand. The physical appearances and sizes of both CO and SO were shown in Supplementary Table S1 and S2, respectively.

Fresh samples were cleaned with deionized (DI) water before separating flesh from seed. The color of fresh samples was analyzed using a ColorFlex EZ spectrophotometer from Hunter Associates Laboratory (Reston, VA, USA). The results were expressed as CIELAB units with L* representing dark (0) to white (100) colors, a* representing green (−) to red (+) colors, and b* representing blue (−) to yellow (+) colors as shown in Supplementary Table S3. Clean samples were cut into small pieces (approximately 0.3 cm thick), freeze dried for 3 days using a freeze dryer (a Heto powerdry PL9000 from Heto Lab Equipment, Allerod, Denmark), and ground into fine powder using a grinder (a Philips 600W series from Philips Electronic Co., Ltd., Jakarta, Indonesia). The sizes of powdery samples were in the range of 25–35 mesh. The moisture contents of dry samples were analyzed by a Halogen moisture analyzer (a HE53 series from Mettler-Toledo AG, Greifensee, Switzerland) and expressed as percentage of moisture content. The powdery samples were kept at −20 °C until analysis.

The extraction of date palm fruits was optimized as previously described [54]. Briefly, the powdery samples (1 g) were dissolved in distilled water (10 mL), incubated in a 50°C for 1 h using a temperature controlled water bath shaker (a WNE45 series from Memmert GmBh, Eagle, WI, USA), and centrifuged at 3800× *g* for 15 min using a refrigerated centrifuge (a Hettich^®^ ROTINA 38R from Andreas Hettich GmbH, Tuttlingen, Germany). The supernatant was collected and filtered through a 0.45 µM polyether sulfone (PES) membrane syringe filter. The extracted samples were stored at −20 °C until analysis.

### 4.2. Determination of Nutritive Values

Nutritive values of fresh samples were analyzed, including energy, protein, fat, carbohydrate, fiber, sugar, ash, moisture content, vitamins (A, D, E, and C) and minerals (Ca, P, Na, K, Mg, Fe, and Zn). The standard protocols of the Association of Official Analytical Chemists (AOAC) [55] were employed according to the previous report [56,57]. All experiments were performed at the Institute of Nutrition, Mahidol University with the international standard for laboratory quality systems (ISO/IEC 17025:2005). The results were reported as per 100 g fresh weight (FW) as shown in Supplementary Table S4. However, these results were calculated and presented as per 100 g dry weight (DW) to accurately compare the effect of plant origins and fruit parts on nutritive values.

The moisture content was investigated using the drying method (AOAC 930.04, 934.01), while protein content was analyzed using the Kjeldahl method (AOAC 992.23). Fat content was determined through acid hydrolysis and petroleum ether extraction (AOAC 948.15, 945.16), and ash content was calculated by incineration (AOAC 930.30, 945.46). Total carbohydrate content and energy were calculated using the following equations:

Total carbohydrate (g) = 100 − Moisture content (g) − Protein (g) − Fat (g) − Ash (g)(1)

Energy (kcal) = [Protein (g) × 4] + [Carbohydrate (g) × 4] + [Fat (g) × 9](2)

Total dietary fiber was calculated by the sum of soluble and insoluble dietary fibers, which were determined according to AOAC 993.19 and 991.42, respectively.

Disaccharides (fructose, glucose, sucrose, and maltose) were analyzed using an ultra-fast liquid chromatographic (UFLC) system (Shimadzu Corporation, Kyoto, Japan) with an Alltech 800 evaporative light scatteringdetector (ELSD) (BUCHI Corporation, New Castle, DE, USA). The sample was separated on a Shodex Asahi Pak NH2P-50 4E column (5 µm, 250 × 4.6 mm from Shodex Group, Kanagawa, Japan) with an isocratic solvent system (76% (*v*/*v*) acetonitrile) and a flow rate of 1.0 mL/min.

Vitamin A was determined after alkaline saponification and organic extraction as β-carotene, which is separated from other carotenoids based on previous literature with modifications as follows [58]. The powdered sample (0.5 g) was mixed with ascorbic acid solution (10 mL) and 2N KOH (50 mL) before refluxing on a boiling water bath (Memmert GmBh, Eagle, WI, USA) for 30 min. The mixture was cooled on ice before adding hexane (70 mL). The upper layer of the mixture was removed into a separating funnel that contained 5% (*w*/*v*) KOH solution (50 mL) and re-extracted with hexane (35 mL) twice. The upper layers were combined and washed with 10% (*w*/*v*) NaCl solution (100 mL) and then DI water (100 mL) three times. The extract was evaporated and redissolved with minimum volume of methanol before filtrating through a 0.2 µm polytetrafluoroethylene (PTFE) syringe filter. The filtrate was loaded onto a 5 µm C18 column (90Å, 150 × 4.6 mm from Waters Corporation, Milford, MA, USA) connecting to the reverse phase—high performance liquid chromatography (HPLC) utilizing a Shimadzu LC-20AD pump (Shimadzu Corporation, Kyoto, Japan) and a UV/Vis detector (UV-975, JASCO International Co., Ltd., Tokyo, Japan). The isocratic mobile phases composing of absolute methanol with a constant flow rate of 0.6 mL/min. The existence of vitamin A was visualized at 325 nm by comparing retention time (*t_R_*) and spectral fingerprint with a β-carotene standard.

Vitamin E was analyzed using the set-up protocol similarly to those of vitamin A. However, the existence of vitamin E was visualized at 294 nm by comparing *t_R_* and spectral fingerprint with a tocopherol standard.

Vitamin C content was determined using a well-established protocol in the ASEAN manual of Food Analysis, 2011 [59]. Briefly, the sample was extracted using 10% (*v*/*v*) metaphosphoric acid (MPA), and vitamin C was identified using a Zorbax original ODS column (5 µm, 250 × 4.6 mm from Agilent Technologies, Santa Clara, CA, USA) on the HPLC system utilizing a Waters 515 pump (Waters Corporation, Milford, MA, USA), a UV/Vis detector (UV-975, JASCO International Co., Ltd., Tokyo, Japan), an isocratic solvent system (0.5% (*v*/*v*) KH_2_PO_4_, adjusted pH to 2.5 with H_3_PO_4_), and a flow rate of 0.8 mL/min. The existence of vitamin C was monitored at 254 nm.

Vitamin D was analyzed by HPLC system according to AOAC 995.05. The detection of vitamin D was performed utilizing a Vydac C18 column (5 µm, 205 × 4.6 mm from Grace Davison Discovery Sciences, Rowville, VIC, Australia) on an Agilent 1100 HPLC system with a photodiode array detector (Agilent Technologies, Santa Clara, CA, USA). The existence of vitamin D was visualized at 265 nm by comparing *t_R_* and spectral fingerprint with a vitamin D2 standard.

The analysis of calcium, sodium, and potassium contents were performed under an Atomic Absorption Spectrometer (AAS) (S Series, Thermo Electron Corporation, Cambridge, UK) (AOAC 985.35). Phosphorus content was determined using the gravimetric method [60], while magnesium, zinc, and iron contented were analyzed utilizing an Inductively Coupled Plasma Optical Emission Spectrophotometer (ICP-OES) (an Optima 4200DV from PerkinElmer^®^, Waltham, MA, USA) (AOAC 984.27).

### 4.3. Determination of Phenolic and Carotenoid Profiles

To determined phenolic profiles of flesh and seeds of CO and SO, the samples were extracted using acidic methanol as previously described [61]. The extracted were then injected into HPLC system utilizing an Agilent 1100 HPLC with a photodiode array detector and a Zorbax Eclipse XDB-C_18_ column (5 µm, 150 × 4.6 mm from Agilent Technologies) with a gradient mobile phases (solvent A: Milli-Q water (18.2 MΩ.cm resistivity at 25 °C) containing 0.05% (*v*/*v*) trifluoroacetic acid (TFA), solvent B: methanol containing 0.05% (*v*/*v*) TFA, and solvent C: acetonitrile containing 0.05% (*v*/*v*) TFA) and a constant flow rate of 0.6 mL/min (Table 4). The existence of phenolics was confirmed by comparing *t_R_* and spectral fingerprint with standards using an Agilent ChemStation software. The standards of phenolic acids including caffeic acid (> 98.0% HPLC, T), chlorogenic acid (> 98.0% HPLC, T), *p*–coumaric acid (> 98.0% GC, T), 3,4–dihydroxybenzoic acid (≥ 97% T), 4–hydroxybenzoic acid (> 99.0% GC, T), ferulic acid (> 98.0% GC, T), syringic acid (> 97.0% T), and sinapic acid (>99.0% GC, T) were obtained from Tokyo Chemical Industry (Tokyo, Japan), while gallic acid (97.5–102.5% T), rosmarinic acid (≥ 98% HPLC), and vanillic acid (≥ 97% HPLC) were received from Sigma–Aldrich (St. Louis, MO, USA). The standards of flavonoids including quercetin (> 98.0% HPLC, E), kaempferol (> 97.0% HPLC), genistein (> 98.0% HPLC), luteolin (> 98.0% HPLC), hesperidin (> 90.0% HPLC, T), naringenin (> 93.0% HPLC, T), myricetin (> 97.0% HPLC), and apigenin (> 98.0% HPLC) were from Tokyo Chemical Industry (Tokyo, Japan), while rutin (≥ 94% HPLC) was received from Sigma–Aldrich and isorhamnetin (≥ 99.0% HPLC) was obtained from Extrasynthese (Genay, France). The phenolic acids were visualized at 280 and 325 nm, while flavonoids were visualized at 280, 338, and 368 nm. The HPLC chromatograms were shown in Supplementary Figure S1–S4.

Total phenolic contents (TPCs) and total flavonoid contents (TFCs) were analyzed utilizing a well-established protocol as previously described [62,63]. TPCs used Folin–Ciocalteu’s phenol as a reagent and gallic acid (0–200 µg/mL) as a standard. Likewise, TFCs were analyzed using aluminum trichloride as a reagent and quercetin (0–100 µg/mL) as a standard.

Carotenoid profiles were analyzed according to the previous report [58] with some modifications as follows. The powdered sample (0.3 g) was mixed with phosphate buffered saline (PBS) (2 mL) and homogenized 3 times (30 s/time). To the extractant, the mixture of methanol and tetrahydrofuran (THF) in a ratio of 1:1 (5 mL) was added and vortexed for 1 min. The mixture was sonicated in an ultrasonic bath for 2 min. Hexane (5 mL) was then added into the mixture before vortexing for 2 min and centrifuging for 10 min at 3800 × *g* at 25 °C (a Hettich^®^ ROTINA 38R centrifuge from Andreas Hettich GmbH, Tuttlingen, Germany). After that, the upper layer of the mixture was removed into the bottom flask and re-extracted with hexane until the sample was colorless (2 times). The solvent was removed by evaporation at 37 °C, and the remaining residue was re-dissolved with the mixture of methyl tert-butyl ether (MtBE) and methanol in a ratio of 3:1 (1.5 mL) and methanol (0.5 mL), respectively. The extract was then filtrated through a 0.2 µm PTFE syringe filter before loading onto a Vydaс 201ТР-C18 column (5 µm, 150 × 4.6 mm from Alltech Associates, Inc., Columbia, MD, USA), which connected to an Agilent 1100 HPLC system and a photodiode array detector (Agilent Technologies). The gradient mobile phases composing of absolute MtBE (solvent A) and methanol containing 2% (*v*/*v*) ammonium acetate (solvent B) with a constant flow rate of 0.6 mL/min at 25 °C were shown in Table 5. The existence of carotenoids were visualized at 450 nm by comparing *t_R_* and spectral fingerprint with the standards, including lutein (>96.0% HPLC), zeaxanthin (> 95.0% HPLC), β-cryptoxanthin (> 97.0% TLC), α-carotene (>95.0% HPLC), β-carotene (> 95.0% HPLC), and lycopene (> 98.0% HPLC) from Sigma-Aldrich. The HPLC chromatograms were shown in Supplementary Figure S5.

### 4.4. Determination of Enzyme Inhibitory Activities

The inhibitory activities of the key enzymes that control some non-communicable diseases including obesity (lipase), diabetes (α-amylase, α-glucosidase, and dipeptidyl peptidase-IV), Alzheimer’s disease (acetylcholinesterase, butyrylcholinesterase, and β-secretase), and hypertension (angiotensin-converting enzyme) were determined using a well-established protocol as previously described [57,64]. The enzyme inhibitory assays were consisted of an enzyme, a substrate, and an indicator from Sigma-Aldrich (St. Louis, MO, USA), and a sample extract as shown in Table 6. The assay was monitored using a Synergy^TM^ HT 96-well UV-visible microplate reader and a Gen5 data analysis software (BioTek Instruments, Inc., Winooski, VT, USA).

The inhibitory results using enzyme kinetics were expressed as inhibition percentage using the following equation:

(3) $$\%\;\mathrm{inhibition}\;=\;\left(1-\;\;\frac{B-b}{A-a}\right)\;\times \;100$$

where *A* is the initial velocity of the control reaction with enzyme (control), *a* is the initial velocity of the control reaction without enzyme (control blank), *B* is the initial velocity of the enzyme reaction with extract (sample), and *b* is the initial velocity of the reaction with extract but without enzyme (sample blank). The inhibitory results using enzyme end-point assay were expressed as inhibition percentage using the same equation, but changing from initial velocity to absorbance at particular wavelength.

### 4.5. Statistical Analysis

All experiments were carried out in triplicate (n = 3) and expressed as mean ± standard deviation (SD). Unpaired t-test was performed to determine the significant differences between values with *p* < 0.05.

## 5. Conclusions 

Results obtained from this study provide information regarding the effects of planting techniques and parts of date palm fruits on nutritive values, bioactive compounds, antioxidant activities, and medicinal abilities against obesity, diabetes, hypertension, and Alzheimer’s disease. Comparison between CO and SO fruits showed that CO contained higher total carbohydrate, sugar, and Fe contents, while SO contained higher protein, total dietary fiber (especially IDF), vitamin A, vitamin E, P, Ca, Na, Mg, and Zn contents. SO also contained higher phenolic contents, leading to greater enzyme inhibitory activities. Thus, consumers aiming for sweetness should choose CO fruits. However, less sweet SO fruits are more nutritious with greater health benefits. This information can be used to promote consumption of seed originated date palm fruit to reduce agricultural cost and develop date palm fruit as new products. Date palm seed also requires further in vivo study and development to assess its suitability as a by-product with valuable bioactive compounds and potential food applications. Our results suggested that date palm seeds could be utilized for future food development.

## Figures and Tables

**Table 1:** Nutritive values of flesh and seed of cell culture originated (CO) and seed originated (SO) date palm fruits at Khalal stage of Barhi cultivar.

Nutrients	Nutritive Value (Per 100 g Dry Weight)			
	Flesh		Seed	
	CO	SO	CO	SO
**Energy (kcal)**	388.07 ± 2.41 **	385.13 ± 0.76 **	441.21 ± 2.42 *	432.85 ± 1.73
**Protein (g)**	2.68 ± 0.08 *,**	4.64 ± 0.14 **	4.88 ± 0.09 *	6.09 ± 0.13
**Fat (g)**	0.19 ± 0.04 **	0.00 ± 0.00 **	9.24 ± 0.49 *	7.73 ± 0.35
**Total carbohydrate (g)**	93.47 ± 0.35 *,**	91.64 ± 0.27 **	84.64 ± 0.53	84.73 ± 0.32
**Total dietary fiber (g)**	16.62 ± 0.44 *,**	23.44 ± 1.34 **	84.14 ± 2.95	81.95 ± 0.98
**SDF**	2.37 ± 0.04 **	4.13 ± 0.97 **	1.08 ± 0.47	1.55 ± 0.04
**IDF**	14.24 ± 0.44 *,**	19.30 ± 0.60 **	83.05 ± 2.88	80.41 ± 0.99
**Ash (g)**	3.46 ± 0.29 **	3.72 ± 0.19 **	1.25 ± 0.06 *	1.45 ± 0.02
**Total sugar (g)**	74.25 ± 2.37 *,**	62.50 ± 2.77 **	3.60 ± 0.04	3.58 ± 0.13
**Fructose**	35.63 ± 1.26 *,**	29.57 ± 1.73 **	1.87 ± 0.08	1.83 ± 0.11
**Glucose**	36.53 ± 1.14 *,**	32.93 ± 1.04 **	1.73 ± 0.09	1.75 ± 0.04
**Sucrose**	1.58 ± 0.08 *,**	0.00 ± 0.00	0.00 ± 0.00	0.00 ± 0.00
**Maltose**	0.52 ± 0.03 *,**	0.00 ± 0.00	0.00 ± 0.00	0.00 ± 0.00
**Vitamins**				
**Vitamin A (µg)**	10.06 ± 0.18 *,**	30.19 ± 0.93 **	1.62 ± 1.14	2.20 ± 2.33
**Vitamin D2 (µg)**	<LOD	<LOD	<LOD	<LOD
**Vitamin E (mg)**	0.59 ± 0.03 *,**	23.64 ± 0.88 **	0.06 ± 0.00	0.07 ± 0.01
**Vitamin C (mg)**	ND	ND	ND	ND
**Minerals (mg)**				
**Calcium**	63.92 ± 13.69 *,**	107.12 ± 3.99 **	30.25 ± 0.64 *	34.38 ± 1.22
**Phosphorus**	115.04 ± 6.43 *,**	185.28 ± 38.09	156.33 ± 12.26	182.24 ± 13.65
**Sodium**	52.75 ± 3.00 *,**	74.94 ± 6.68 **	20.21 ± 4.92	16.24 ± 8.56
**Potassium**	1051.56 ± 68.09 **	1091.53 ± 52.52 **	321.79 ± 55.1	312.16 ± 38.48
**Magnesium**	44.36 ± 2.26 *,**	49.74 ± 1.00 **	68.73 ± 1.61 *	74.81 ± 1.16
**Iron**	0.87 ± 0.05 *,**	0.72 ± 0.08 **	1.06 ± 0.05	1.15 ± 0.20
**Zinc**	0.29 ± 0.05 *,**	0.46 ± 0.06	0.84 ± 0.02	0.55 ± 0.41

All data were expressed as mean ± standard deviation (SD) of triplicate experiments (n = 3). SDF: soluble dietary fiber; IDF: insoluble dietary fiber; ND: not detected; vitamin A presented as µg β-carotene; vitamin E presented as mg α-tocopherol; < LOD: Limit of detection at 0.05 µg/100 g; * showed significant difference (*p* < 0.05) between values in the same fruit part of cell culture originated (CO) and seed originated (SO) date palm fruit using unpaired t-test; ** showed significant difference (*p* < 0.05) between values in flesh and seed of the same originated date palm fruit using unpaired t-test.

**Table 2:** Total phenolic contents (TPCs) and total flavonoid contents (TFCs), phenolic profiles (phenolic acids and flavonoids), and carotenoid profiles of flesh and seed of cell culture originated (CO) and seed originated (SO) date palm fruits at Khalal stage of Barhi cultivars.

Bioactive Compounds	Flesh		Seed	
	CO	SO	CO	SO
**TPCs (mg GAE/g DW)**	2.59 ± 0.11 *,**	3.94 ± 0.17 **	10.76 ± 0.97 *	18.41 ± 1.83
**TFCs (mg QE/g DW)**	1.95 ± 0.17 *,**	4.01 ± 0.15 **	7.43 ± 0.30 *	13.68 ± 1.14
**Phenolic acids (mg per 100 g DW)**				
**Gallic acid**	ND	ND	0.70 ± 0.04	0.74 ± 0.03
**Vanillic acid**	6.12 ± 0.38 *,**	8.19 ± 0.20 **	7.00 ± 0.13 *	4.94 ± 0.35
**Caffeic acid**	0.16 ± 0.00 *,**	3.56 ± 0.10 **	3.31 ± 0.04	3.27 ± 0.06
**Syringic acid**	4.11 ± 0.35 *	3.19 ± 0.10	3.84 ± 0.06 *	3.18 ± 0.10
***p*** **-Coumaric acid**	1.80 ± 0.02 *,**	1.52 ± 0.07 **	1.35 ± 0.16	1.15 ± 0.04
**Ferulic acid**	7.91 ± 0.10 *,**	9.71 ± 0.19 **	1.75 ± 0.03 *	1.11 ± 0.02
**Sinapic acid**	2.06 ± 0.15 *,**	6.60 ± 0.06 **	0.51 ± 0.02 *	0.36 ± 0.02
**Flavonoids (mg per 100 g DW)**				
**Hesperidin**	4.72 ± 0.10 *	3.53 ± 0.04	ND	ND
**Luteolin**	0.71 ± 0.00 *,**	1.93 ± 0.01 **	0.94 ± 0.02 *	1.14 ± 0.05
**Quercetin**	ND	ND	1.05 ± 0.01 *	2.48 ± 0.07
**Kaempferol**	3.62 ± 0.03 *,**	2.13 ± 0.02 **	0.40 ± 0.01 *	0.56 ± 0.02
**Apigenin**	0.80 ± 0.02 *,**	0.36 ± 0.01 **	0.31 ± 0.00	0.32 ± 0.02
**Isorhamnetin**	4.51 ± 0.08 *,**	5.76 ± 0.03 **	2.93 ± 0.03 *	4.03 ± 0.02
**Carotenoids (mg per 100 g DW)**				
**Lutein**	205.96 ± 5.01 *	1076.00 ± 25.68	ND	ND
**β-Cryptoxanthin**	51.76 ± 2.11 *	99.31 ± 6.93	ND	ND
**α-Carotene**	153.81 ± 1.29 *	298.80 ± 4.11	ND	ND
**β-Carotene**	96.15 ± 1.87 *	218.90 ± 6.78	ND	ND

All data were expressed as mean ± standard deviation (SD) of triplicate experiments (n = 3). GAE: gallic acid equivalent; QE: quercetin equivalent; DW: dry weight; ND: not detected; * showed significant difference (*p* < 0.05) between values in the same fruit part of cell culture originated (CO) and seed originated (SO) date palm fruits using unpaired t-test; ** showed significant difference (*p* < 0.05) between values in flesh and seed of the same originated date palm fruit using unpaired t-test.

**Table 3:** Enzyme inhibitory activities of flesh and seed of cell culture originated (CO) and seed originated (SO) date palm fruits at Khalal stage of Barhi cultivars.

Enzyme Reactions	Enzyme Inhibitory Activities (%Inhibition)			
	Flesh		Seed	
	CO	SO	CO	SO
**^1^** **Lipase**	5.76 ± 0.24 *,**	4.72 ± 0.38 **	20.66 ± 1.61 *	77.14 ± 7.51
**^2^** **α** **-Amylase**	ND	ND	63.51 ± 3.66 *	70.14 ± 3.09
**^3^** **α** **-Glucosidase**	50.52 ± 5.03 *	44.64 ± 0.87	60.38 ± 5.81 *	52.33 ± 5.03
**^4^** **DPP-IV**	ND	4.39 ± 0.56	ND	ND
**^5^** **AChE**	ND	23.49 ± 1.60	67.97 ± 4.56 *	49.49 ± 3.29
**^6^** **BChE**	ND	31.20 ± 3.05	46.80 ± 3.34 *	27.19 ± 1.04
**^7^** **BACE-1**	75.05 ± 1.25 *,**	70.73 ± 0.44 **	39.23 ± 3.52 *	24.50 ± 2.05
**^8^** **ACE**	20.68 ± 0.60 *,**	85.07 ± 0.30 **	58.69 ± 0.93	61.36 ± 4.95

All data are expressed as mean ± standard deviation (SD) of triplicate experiments (n = 3). DPP-IV: dipeptidyl peptidase-IV; AChE: acetylcholinesterase; BChE: butyrylcholinesterase; BACE-1: β-secreatse; ACE: angiotensin-converting enzyme; ND: not detected; ^1^concentration of flesh and seed extracts = 2.5 mg/mL; ^2^concentration of flesh and seed extracts = 25 mg/mL; ^3^concentration of flesh extract = 2.5 mg/mL and of seed extracts = 0.075 mg/mL; ^4^concentration of flesh and seed extracts = 12.5 mg/mL; ^5^concentration of flesh extract = 20 mg/mL and of seed extracts = 2 mg/mL; ^6^concentration of flesh extract = 20 mg/mL and of seed extracts = 0.2 mg/mL; ^7^concentration of flesh and seed extracts = 20 mg/mL; ^8^concentration of flesh and seed extracts = 0.55 mg/mL; * showed significant difference (*p* < 0.05) between values in the same fruit part of cell culture originated (CO) and seed originated (SO) date palm fruits using unpaired t-test; ** showed significant difference (*p* < 0.05) between values in flesh and seed of the same originated date palm fruit using unpaired t-test.

**Table 4:** High performance liquid chromatography (HPLC) conditions to determine phenolics.

Time (min)	Flow Rate (mL/min)	Solvent A (%)	Solvent B (%)	Solvent C (%)
0	0.6	90	6	4
5	0.6	85	9	6
30	0.6	71	17.4	11.6
60	0.6	0	85	15
61	0.6	90	6	4
66	0.6	90	6	4

Solvent A = Milli-Q water containing 0.05% (*v*/*v*) trifluoroacetic acid (TFA); solvent B = methanol containing 0.05% (*v*/*v*) TFA; solvent C = acetonitrile containing 0.05% (*v*/*v*) TFA.

**Table 5:** High performance liquid chromatography (HPLC) conditions to determine carotenoids.

Time (min)	Flow rate (mL/min)	Solvent A (%)	Solvent B (%)
0	0.6	20	80
1	0.6	20	80
10	0.6	40	60
20	0.6	60	40
25	0.6	75	25
30	0.6	75	25
32	0.6	20	80
37	0.6	20	80

Solvent A = absolute methyl tert-butyl ether (MtBE); solvent B = methanol containing 2% (*v*/*v*) ammonium acetate.

**Table 6:** The compositions, types of assay, and detecting wavelengths of the enzyme inhibitory assays.

Assay	Enzyme	Substrate	Indicator	Inhibitor	Assay Type	Detecting Wavelength
Lipase	100 µL of 0.01 mg/mL *Candida rugosa* lipase (type VII, ≥700 unit/mg)	50 µL of 0.2 mM 5-5′-dithiobis(2-nitrobenzoic-*N*-phenacyl-4,5-dimethyyhiazolium bromide)	10 µL of 16 mM DTNB	40 µL of extract	Kinetics	412 nm
α-Amylase	100 µL of 30 mg/mL porcine pancreatic α-amylase (type VII, ≥ 10 unit/mg)	50 µL of 30 mM *p*-nitrophenyl-α-d-maltopentaoside		50 µL of extract	Kinetics	405 nm
α-Glucosidase	100 µL of 0.1 U/mL *Saccharomyces cerevisiae* α-glucosidase (type 1, ≥10 U/mg protein)	50 µL of 2 mM *p*-nitrophenyl-α-D-glucopyranoside		50 µL of extract	Kinetics	405 nm
DPP-IV	100 µL of 0.01 U/mL human dipeptidyl peptidase-IV (recombinant, ≥ 10 units/mg)	50 µL of 6 mM Gly-Pro-*p*-nitroanilide hydrochloride		50 µL of extract	Kinetics	405 nm
AChE	100 μL of 20 ng *Electrophorus electricus* AChE (1,000 units/mg)	40 μL of 0.8 mM acetylthiocholine	10 µL of 16 mM DTNB	40 µL of extract	Kinetics	412 nm
BChE	100 µL of 0.5 µg/mL equine serum BChE (≥ 10 units/mg)	50 µL of 0.4 mM butyrylthiocholine	10 µL of 16 mM DTNB	40 µL of extract	Kinetics	412 nm
BACE-1	a BACE-1 FRET assay kit			20 µL of extract	End-point	λ_ex_ = 320 nm, λ_em_ = 405 nm
ACE	3 µL of 0.5 U/mL rabbit lung ACE (≥ 2 unit/mg)	30 µL of 3 mM hippuryl-histidyl-leucine	15 µL of 20 mg/mL PDA	50 µL of extract	End-point	λ_ex_ = 360 nm, λ_em_ = 485 nm

DPP-IV: dipeptidyl peptidase-IV; AChE: acetylcholinesterase; BChE: butyrylcholinesterase; BACE-1: β-secretase; ACE: angiotensin-converting enzyme; FRET: fluorescence resonance energy transfer; DTNB: 5,5′-dithiobis(2-nitrobenzoic acid); PDA: *o*-phthaldialdehyde.

## Data Availability

Data is contained within this article and supplementary material.

## Supplementary Materials

Supplementary Table S1. Images of whole bunch, sectioned fruit, flesh (mesocarp + epicarp), and seed of cell culture originated (CO) and seed originated (SO) date palm fruits at Khalal stage of Barhi cultivars; Supplementary Table S2. Size (width x length x thickness) in centimeters of flesh (mesocarp + epicarp) and seed of cell culture originated (CO) and seed originated (SO) date palm fruits at Khalal stage of Barhi cultivars; Supplementary Table S3. Color (where L* representing dark (0) to white (100) colors, a* representing green (-) to red (+) colors, and b* representing blue (-) to yellow (+) colors) and the percentage (%) of moisture content of fresh (mesocarp + epicarp) and freeze-dried flesh and seed of cell culture originated (CO) and seed originated (SO) date palm fruits at Khalal stage of Barhi cultivars; Supplementary Table S4. Nutritive values per 100 g fresh weight of flesh and seed of cell culture originated (CO) and seed originated (SO) date palm fruits at Khalal stage of Barhi cultivars; Supplementary Figure S1. High-performance liquid chromatograms of standards including (A.) gallic acid, (B.) vanillic acid, (C.) syringic acid, and (D.) hesperidin and samples including (E.) flesh and (F.) seed of cell culture originated (CO) and (G.) flesh and (H.) seed of seed originated (SO) date palm fruits at Khalal stage of Barhi cultivars. Retention times (*R_t_*) of phenolics in date palm fruit extracts are indicated at a wavelength of 280 nm; Supplementary Figure S2. High-performance liquid chromatograms of standards including (A.) caffeic acid, (B.) *p*-coumaric acid, (C.) ferulic acid, and (D.) sinapic acid, and samples including (E.) flesh and (F.) seed of cell culture originated (CO) and (G.) flesh and (H.) seed of seed originated (SO) date palm fruits at Khalal stage of Barhi cultivars. Retention times (*R_t_*) of phenolics in date palm fruit extracts are indicated at a wavelength of 325 nm; Supplementary Figure S3. High-performance liquid chromatograms of standards including (A.) luteolin, and (B.) apigenin, and samples including (C.) flesh and (D.) seed of cell culture originated (CO) and (E.) flesh and (F.) seed of seed originated (SO) date palm fruits at Khalal stage of Barhi cultivars. Retention times (*R_t_*) of phenolics in date palm fruit extracts are indicated at a wavelength of 338 nm; Supplementary Figure S4. High-performance liquid chromatograms of standards including (A.) quercetin, (B.) kaempferol, and (C.) isorhamnetin and samples including (D.) flesh and (E.) seed of cell culture originated (CO) and (F.) flesh and (G.) seed of seed originated (SO) date palm fruits at Khalal stage of Barhi cultivars. Retention times (*R_t_*) of phenolics in date palm fruit extracts are indicated at a wavelength of 368 nm; Supplementary Figure S5. High-performance liquid chromatograms of standards including (A.) lutein, (B.) β-cryptoxanthin, (C.) α-carotene and (D.) β-carotene and samples including (E.) flesh and (F.) seed of cell culture originated (CO) and (G.) flesh and (H.) seed of seed originated (SO) date palm fruits at Khalal stage of Barhi cultivars. Retention times (*R_t_*) of carotenoids in date palm fruit extracts are indicated at a wavelength of 450 nm.

## Sample Availability

Samples of the compounds are not available from the authors.
