# Supplementary material for: A Comparison of the Nutritional and Biochemical Quality of Date Palm Fruits Obtained Using Different Planting Techniques
Source: Molecules. 2021 Apr 13;26(8):2245. doi: 10.3390/molecules26082245 (PMC8069938; doi:10.3390/molecules26082245)

molecules

MDPI

Supplementary materials

A Comparison of Nutritional and Biochemical Quality of Date Palm Fruits Obtained from Different Planting Techniques

Jeerawan Hinkaew [1], Amornrat Aursalung [1], Yuraporn Sahasakul [1,2], Nattapol Tangsuphoom [1,2], and Uthaiwan Suttisansanee [1,2,*]

1 Institute of Nutrition, Mahidol University, Salaya, Phuttamonthon, Nakhon Pathom 73170, Thailand; ccindy.jee@hotmail.com (J.H.); amornrat.aur@mahidol.ac.th (A.A.); yuraporn.sah@mahidol.ac.th (Y.S.); nattapol.tng@mahidol.ac.th (N.T.)

2 Food and Nutrition Academic and Research Cluster, Institute of Nutrition, Mahidol University, Salaya, Phuttamonthon, Nakhon Pathom 73170, Thailand

* Correspondence: uthaiwan.sut@mahidol.ac.th; Tel.: +662-800-2380 ext. 422

Supplementary Table S1:

Images of whole bunch, sectioned fruit, flesh (mesocarp + epicarp), and seed of cell culture originated (CO) and seed originated (SO) date palm fruits at Khalal stage of Barhi cultivars.

**Fruit parts**	**Physical appearance**	
	**CO**	**SO**
**Whole bunch**	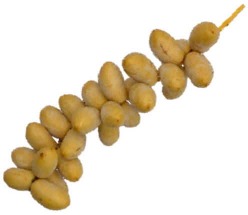	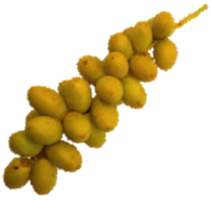
**Sectioned fruit**	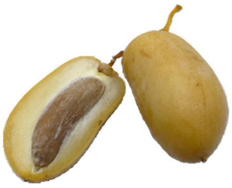	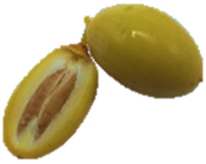
**Flesh**	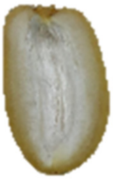	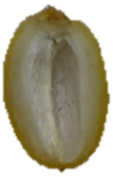
**Seed**	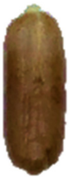	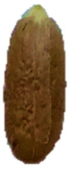

The scale (—) indicated the size of 1 cm.

Supplementary Table S2:

Size (width x length x thickness) in centimeters of flesh (mesocarp + epicarp) and seed of cell culture originated (CO) and seed originated (SO) date palm fruits at Khalal stage of Barhi cultivars.

**Fruit parts**	**Originates**	**Size (cm.)**		
		**Width**	**Length**	**Thickness**
Flesh	CO	2.11 ± 0.10	3.73 ± 0.13	0.67 ± 0.25
	SO	2.00 ± 0.09	3.13 ± 0.30	0.54 ± 0.10
Seed	CO	0.82 ± 0.04	2.55 ± 0.15	0.64 ± 0.04
	SO	0.88 ± 0.04	2.14 ± 0.12	0.61 ± 0.03

All data were expressed as mean ± standard deviation (SD) of triplicate experiments (n = 3).

Supplementary Table S3:

Color (where L* representing dark (0) to white (100) colors, a* representing green (-) to red (+) colors, and b* representing blue (-) to yellow (+) colors) and the percentage (%) of moisture content of fresh and freeze-dried flesh (mesocarp + epicarp) and seed of cell culture originated (CO) and seed originated (SO) date palm fruits at Khalal stage of Barhi cultivars.

**Date palm fruit**	**Color of fresh samples**			**Color of dried samples**			**Moisture content (%) of fresh sample**	**Moisture content (%) of dried sample**
	**L***	**a***	**b***	**L***	**a***	**b***		
**Flesh**								
CO epicarp	48.10 ± 1.39	12.21 ± 0.57	37.28 ± 0.80	56.87 ± 0.74	1.07 ± 0.03	11.72 ± 0.20	68.98 ± 0.69	3.38 ± 0.27
CO mesocarp	65.72 ± 2.99	3.21 ± 0.79	20.85 ± 1.93					
SO epicarp	48.26 ± 4.89	11.80 ± 0.92	46.61 ± 2.65	56.47 ± 0.91	1.14 ± 0.08	13.69 ± 0.60	75.68 ± 0.88	3.30 ± 0.30
SO mesocarp	65.71 ± 1.35	3.87 ± 0.78	21.02 ± 2.05					
**Seed**								
CO	50.30 ± 1.80	6.93 ± 0.24	15.67 ± 0.19	46.70 ± 0.10	4.35 ± 0.12	13.69 ± 0.60	34.42 ± 1.80	3.44 ± 0.62
SO	46.96 ± 1.40	9.97 ± 1.97	21.03 ± 2.78	46.75 ± 0.29	4.56 ± 0.12	13.61 ± 0.27	36.23 ± 0.72	3.41 ± 0.52

All data were expressed as mean ± standard deviation (SD) of triplicate experiments (*n* = 3).

Supplementary Table S4:

Nutritive values per 100 g fresh weight of flesh and seed of cell culture originated (CO) and seed originated (SO) date palm fruits at Khalal stage of Barhi cultivars.

Nutrients	Nutritive value (per 100 g fresh weight)			
	Flesh		Seed	
	CO	SO	CO	SO
**Energy (kcal)**	120.39 ± 3.15 *,**	92.99 ± 3.54 **	289.34 ± 6.41 *	276.04 ± 4.22
**Moisture (g)**	68.98 ± 0.69 *,**	75.86 ± 0.88 **	34.42 ± 1.80	36.23 ± 0.72
**Protein (g)**	0.83 ± 0.02 *,**	1.12 ± 0.02 **	3.20 ± 0.07 *	3.88 ± 0.06
**Fat (g)**	0.12 ± 0.10 **	0.00 ± 0.00 **	6.06 ± 0.16 *	*4.93 ± 0.28
**Total carbohydrate (g)**	29.00 ± 0.74 *,**	22.13 ± 0.87 **	55.51 ± 1.87	54.03 ± 0.46
**Total dietary fiber (g)**	5.15 ± 0.05 **	5.65 ± 0.26 **	55.14 ± 0.42 *	52.26 ± 0.12
- Soluble dietary fiber	0.74 ± 0.02	1.00 ± 0.23	0.71 ± 0.31	0.99 ± 0.03
- Insoluble dietary fiber	4.42 ± 0.05 *,**	4.66 ± 0.04 **	54.43 ± 0.53 *	51.27 ± 0.14
**Ash (g)**	1.07 ± 0.07 *,**	0.90 ± 0.03	0.82 ± 0.05 *	0.92 ± 0.02
**Total sugar (g)**	23.02 ± 0.30 *,**	15.07 ± 0.16 **	2.37 ± 0.06	2.28 ± 0.09
- Fructose	11.05 ± 0.16 *,**	7.13 ± 0.17 **	1.23 ± 0.08	1.17 ± 0.07
- Glucose	11.33 ± 0.15 *,**	7.94 ± 0.05 **	1.14 ± 0.03	1.12 ± 0.04
- Sucrose	0.49 ± 0.03 *,**	0.00 ± 0.00	0.00 ± 0.00	0.00 ± 0.00
- Maltose	0.16 ± 0.01 *,**	0.00 ± 0.00	0.00 ± 0.00	0.00 ± 0.00
**Vitamins**				
- Vitamin A (μg)	3.12 ± 0.02 *,**	7.29 ± 0.26 **	1.08 ± 0.78	1.40 ± 1.48
- Vitamin D2 (μg)	<LOD	<LOD	<LOD	<LOD
- Vitamin E (mg)	0.18 ± 0.01 *,**	5.70 ± 0.27 **	0.04 ± 0.00	0.05 ± 0.01
- Vitamin C (mg)	ND	ND	ND	ND
**Minerals (mg)**				
- Calcium	19.77 ± 3.80	25.86 ± 1.18 **	19.83 ± 0.27 *	21.92 ± 0.61
- Phosphorus	35.70 ± 2.53 **	44.64 ± 8.67 **	102.60 ± 9.87	116.28 ± 9.88
- Sodium	16.38 ± 1.27	18.06 ± 0.99	13.28 ± 3.46	10.37 ± 5.47
- Potassium	326.19 ± 22.26 *,**	263.34 ± 10.00 **	210.69 ± 34.36	199.00 ± 23.82
- Magnesium	13.75 ± 0.40 *,**	12.01 ± 0.46 **	45.06 ± 0.24 *	47.70 ± 0.54
- Iron	0.27 ± 0.01 *,**	0.17 ± 0.02 **	0.69 ± 0.04	0.73 ± 0.13
- Zinc	0.09 ± 0.02 **	0.11 ± 0.01	0.55 ± 0.01	0.35 ± 0.26

All data were expressed as mean ± standard deviation (SD) of triplicate experiments ($n = 3$). Vitamin A presented as μg β-carotene; vitamin E presented as mg α-tocopherol; <LOD: Limit of detection at 0.05 μg/100g; ND: not detect; * showed significant difference ($p < 0.05$) between values in the same fruit part of cell culture originated (CO) and seed originated (SO) date palm fruits using unpaired t-test; ** showed significant difference ($p < 0.05$) between values in flesh and seed of the same originated date palm fruit using unpaired t-test.

Supplementary Figure S1:

High-performance liquid chromatograms of standards including (A.) gallic acid, (B.) vanillic acid, (C.) syringic acid, and (D.) hesperidin and samples including (E.) flesh and (F.) seed of cell culture originated (CO) and (G.) flesh and (H.) seed of seed originated (SO) date palm fruits at Khalal stage of Barhi cultivars. Retention times (R_t) of phenolics in date palm fruit extracts are indicated at a wavelength of 280 nm.

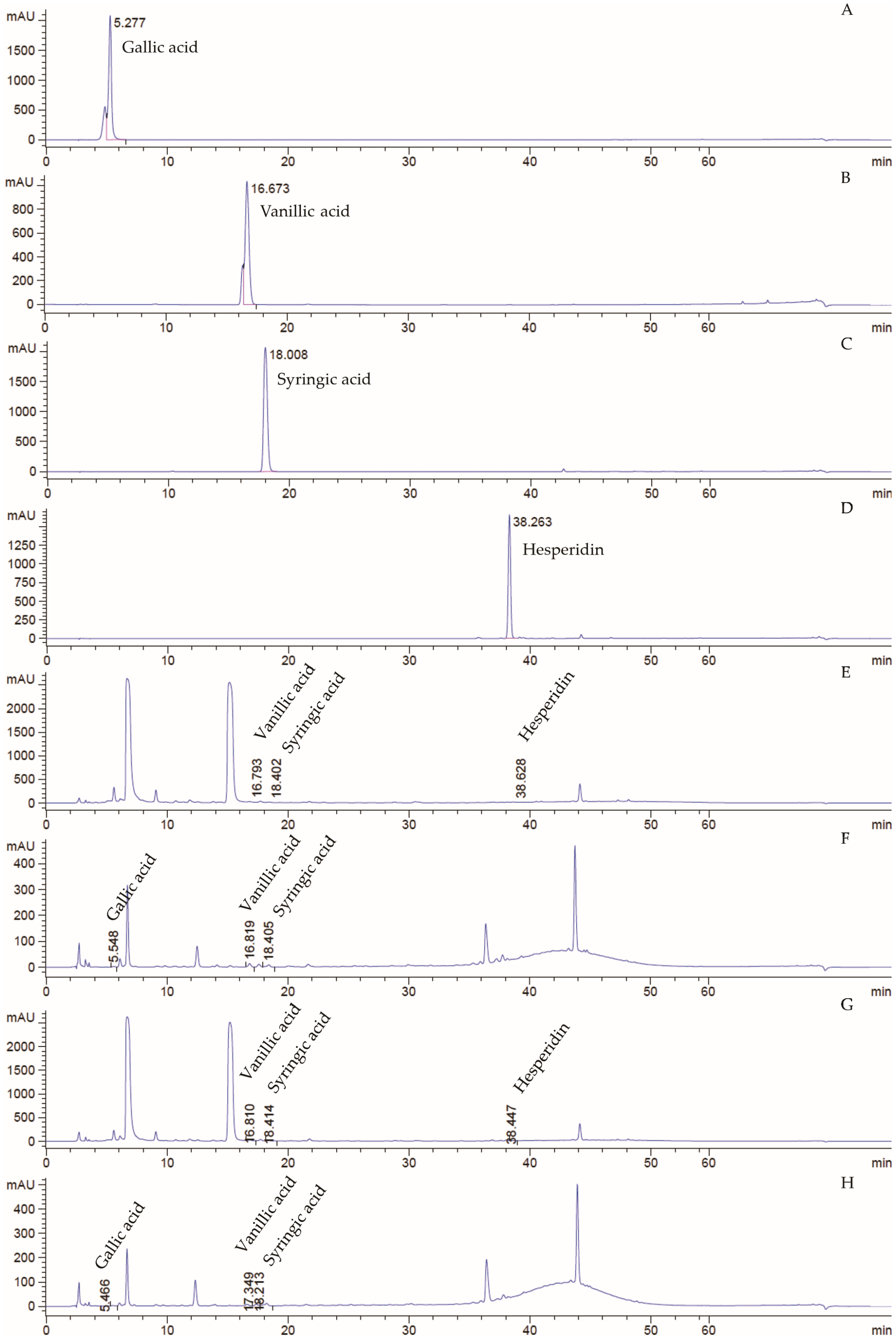

Supplementary Figure S2:

High-performance liquid chromatograms of standards including (A.) caffeic acid, (B.) *p*-coumaric acid, (C.) ferulic acid, and (D.) sinapic acid, and samples including (E.) flesh and (F.) seed of cell culture originated (CO) and (G.) flesh and (H.) seed of seed originated (SO) date palm fruits at Khalal stage of Barhi cultivars. Retention times (R_t) of phenolics in date palm fruit extracts are indicated at a wavelength of 325 nm

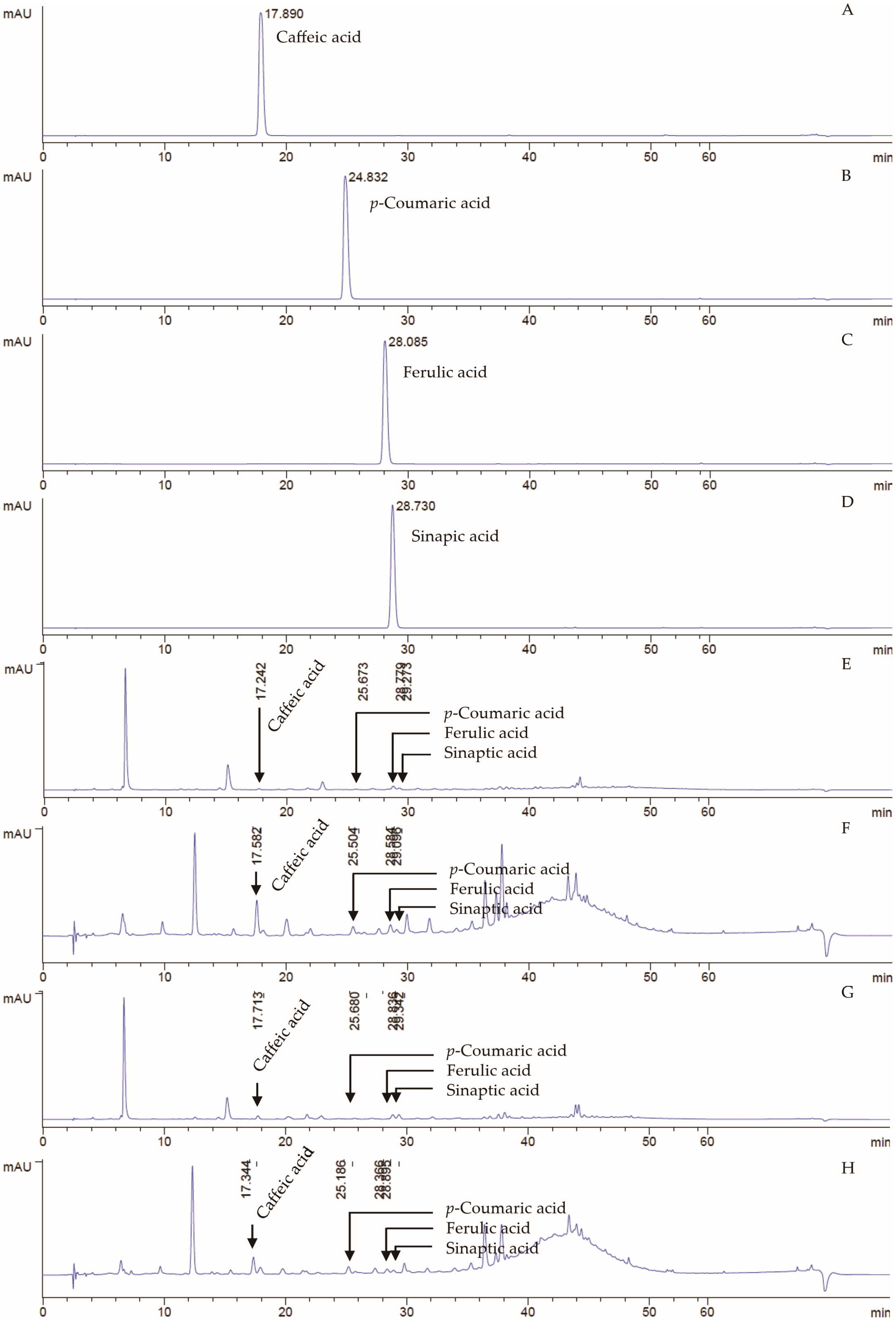

Supplementary Figure S3:

High-performance liquid chromatograms of standards including (A.) luteolin, and (B.) apigenin, and samples including (C.) flesh and (D.) seed of cell culture originated (CO) and (E.) flesh and (F.) seed of seed originated (SO) date palm fruits at Khalal stage of Barhi cultivars. Retention times (R_t) of phenolics in date palm fruit extracts are indicated at a wavelength of 338 nm.

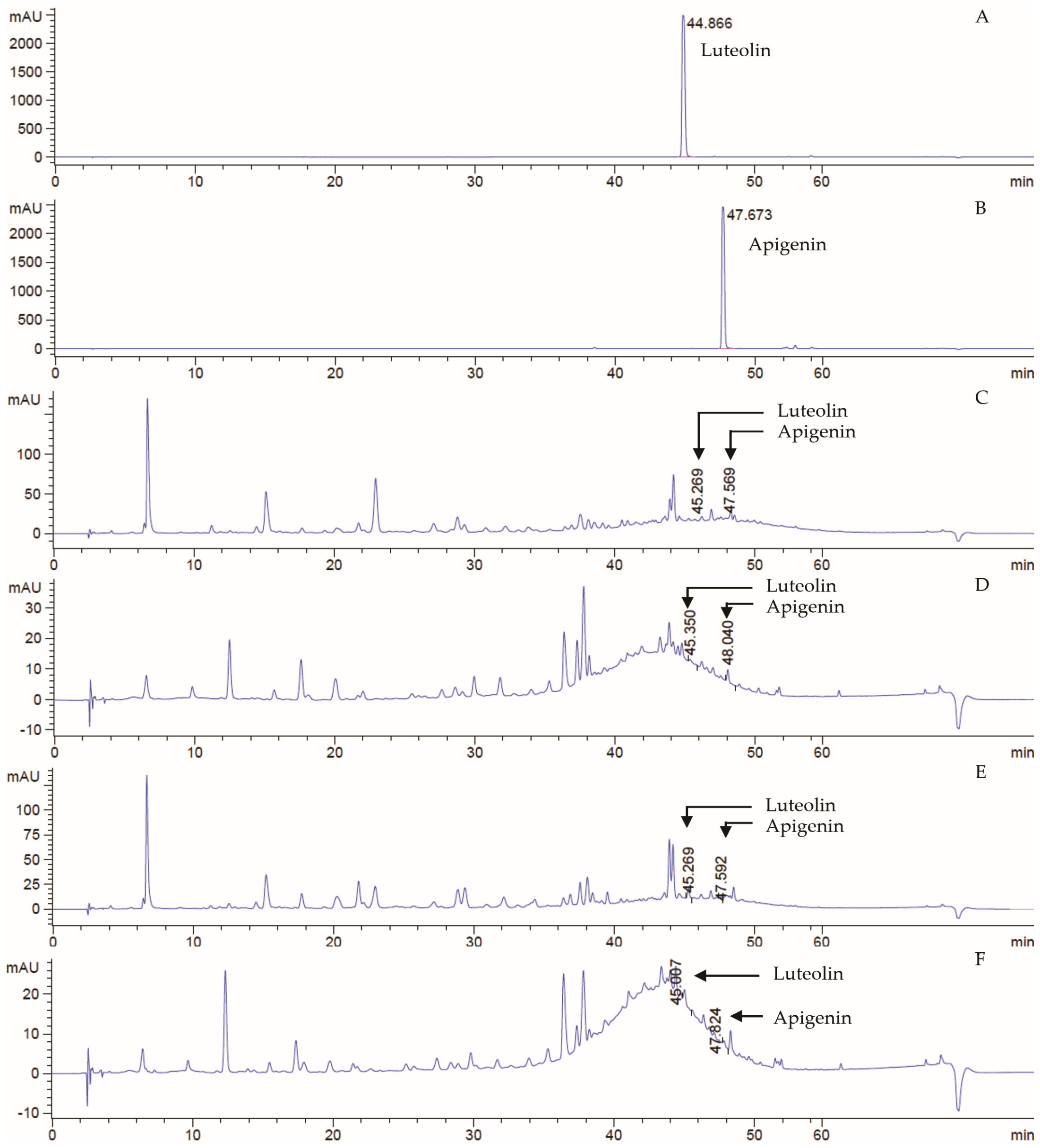

Supplementary Figure S4:

High-performance liquid chromatograms of standards including (A.) quercetin, (B.) kaempferol, and (C.) isorhamnetin and samples including (D.) flesh and (E.) seed of cell culture originated (CO) and (F.) flesh and (G.) seed of seed originated (SO) date palm fruits at Khalal stage of Barhi cultivars. Retention times (R_t) of phenolics in date palm fruit extracts are indicated at a wavelength of 368 nm.

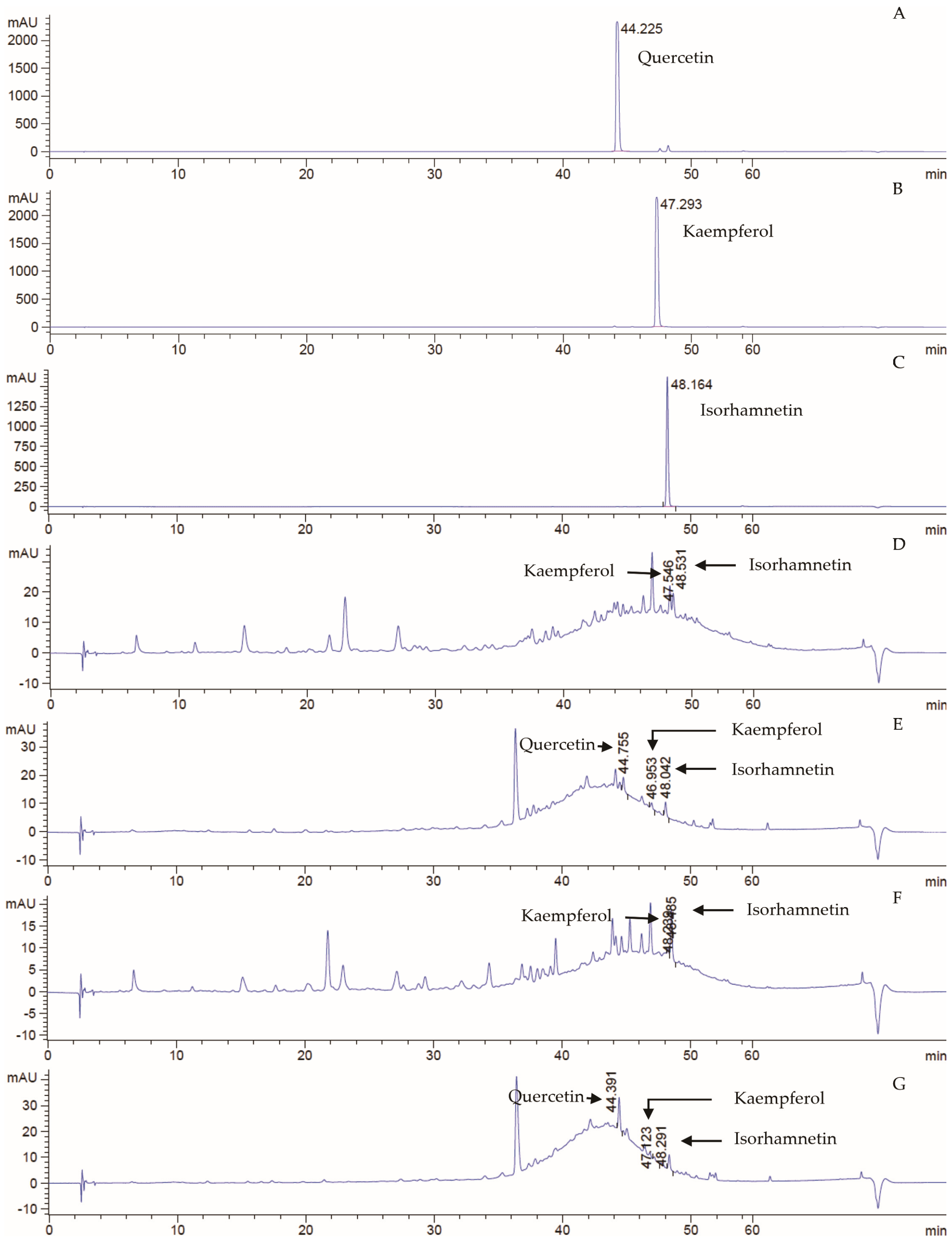

Supplementary Figure S5:

High-performance liquid chromatograms of standards including (A.) lutein, (B.) β-cryptoxanthin, (C.) α-carotene and (D.) β-carotene and samples including (E.) flesh and (F.) seed of cell culture originated (CO) and (G.) flesh and (H.) seed of seed originated (SO) date palm fruits at Khalal stage of Barhi cultivars. Retention times (R_t) of carotenoids in date palm fruit extracts are indicated at a wavelength of 450 nm.

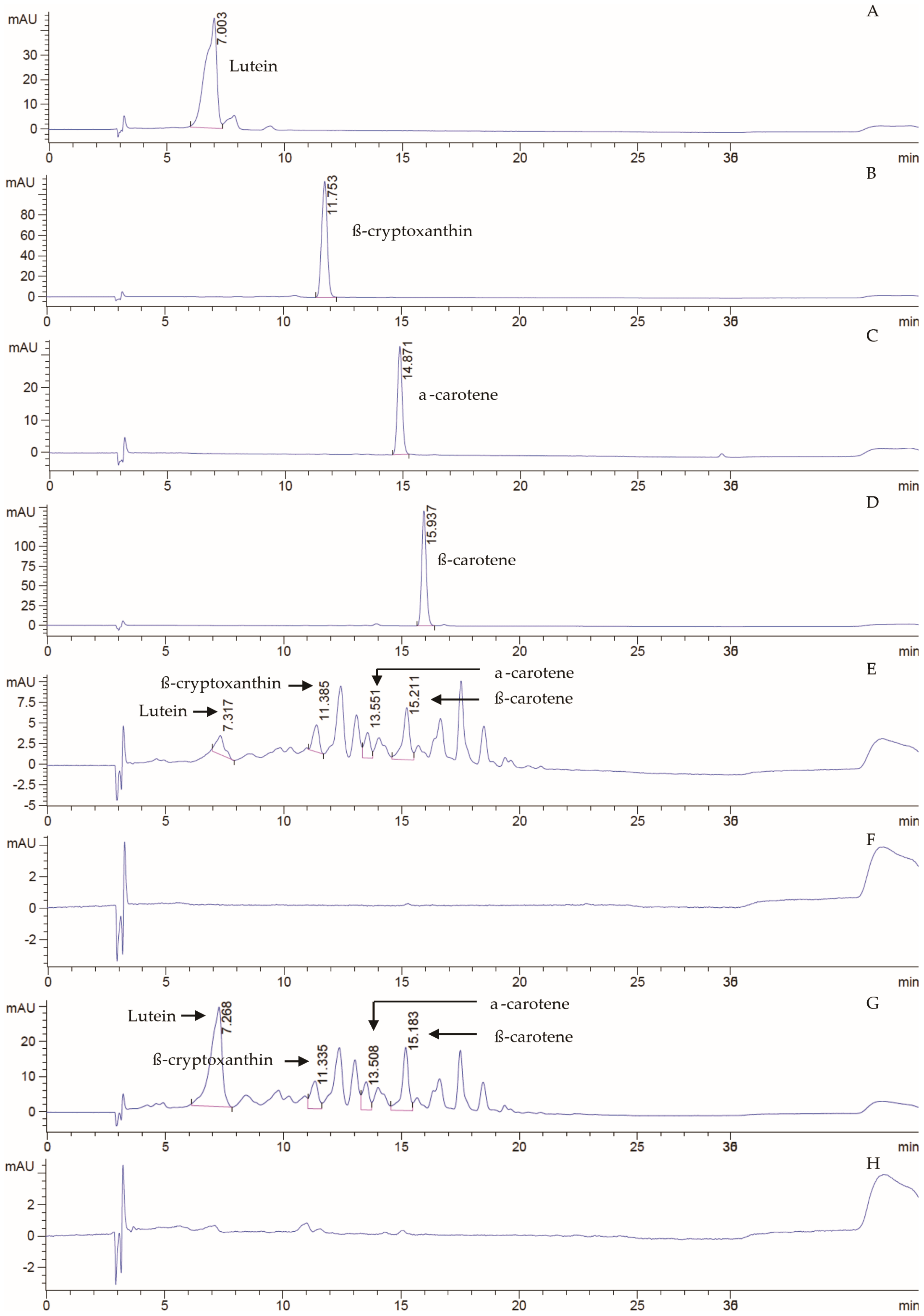

Supplement: Supplementary file 1 [file molecules-26-02245-s001.zip › molecules-1174998-supplementary.pdf]
